# Extranodal marginal zone lymphoma: A case report

**DOI:** 10.1016/j.radcr.2023.09.066

**Published:** 2023-10-19

**Authors:** Walter Camilo Mera Romo, Wilmer Orlando Aponte Barrios, Jorge Alberto Carrillo Bayona, Karen Yuliana Ramírez Iriarte, Luisa Fernanda Gómez Galvis

**Affiliations:** a Diagnostic Imaging Department, Faculty of Medicine, National University of Colombia, National University Hospital of Colombia, Bogotá, Colombia; b Libre University of Santiago of Cali

**Keywords:** Lymphoid neoplasms, Marginal zone lymphoma, Non-Hodgkin's lymphoma, Positron emission tomography (PET/CT), Tomography

## Abstract

Non-Hodgkin lymphomas (NHLs) encompass a diverse range of lymphoproliferative neoplasms. Approximately 85%-90% of NHLs originate from mature B lymphocytes, with the remaining arising from T lymphocytes or natural killer (NK) cells. Notably, NHLs exhibit a pronounced extranodal predilection, with nearly 25% presenting in such locations. In developed countries, the most prevalent NHL subtypes are diffuse large B-cell lymphoma (accounting for 30%) and follicular lymphoma (representing 20%). All other NHL subtypes each constitute less than 10% of cases, including the rarer marginal zone lymphoma (MZL).

We present a case involving a 70-year-old woman who experienced a palpable mass in the right hypochondrium. She displayed no peripheral adenopathies or systemic symptoms. Her diagnosis was established as MZL, posing a diagnostic challenge due to imaging findings that mimicked various infectious, benign, and malignant conditions.

## Introduction

Non-Hodgkin lymphoma (NHL) is a broad and diverse category of lymphomas. According to the World Health Organization (WHO) classification from 2016, NHL encompasses several subtypes, among which is the marginal zone lymphoma (MZL), accounting for 7%-8% of cases. MZL itself can be further subdivided into the extranodal marginal zone lymphoma of mucosa-associated lymphoid tissue (EMZL-MALT), nodal marginal zone lymphoma (NMZL), and splenic marginal zone lymphoma (SMZL) [1], [2], [3], [4], [5].

EMZL, the predominant subtype, represents 50%-70% of all MZLs. Patients diagnosed with EMZL typically have a median age exceeding 60 years. Predominant sites of affliction include the stomach, ocular adnexa, lung, and salivary glands [4].

The etiology of EMZL is often linked with immune cross-reactions. Such reactions can be attributed to chronic exposure to bacterial agents like *Helicobacter pylori, Helicobacter heimanni*, and *Campylobacter jejuni*, viral agents like the hepatitis C virus, or various autoimmune disorders. Approximately 30% of cases are associated with conditions such as Sjögren's syndrome, lymphocytic interstitial pneumonia, multiple sclerosis, rheumatoid arthritis, systemic lupus erythematosus, Hashimoto's thyroiditis, and even antigenic stimulation from chronic smoking [1,2,5].

Clinical manifestations of EMZL vary significantly, contingent upon the organ involved. Symptoms can vary between subtypes. Although some patients might present with non-specific indications like fever, night sweats, and weight loss, these are observed in a mere 5% of cases [2,5].

The role of diagnostic imaging in the assessment of EMZL is pivotal. It assists in characterizing the disease, delineating its extent, guiding biopsies, and facilitating follow-up evaluations.

## Case report

A 70-year-old woman sought medical attention due to a painless mass in her right hypochondrium of 3-week duration. Peripheral adenopathy and systemic symptoms were absent. Her medical history was notable for dyslipidemia, gastroesophageal reflux disease (under treatment), and a smoking history of four cigarettes daily, which she had quit 30 years prior to her diagnosis.

On physical examination, no other masses, adenopathies, or nodules were palpable.

Laboratory tests, including hematology, blood chemistry, liver function, and coagulation, were largely unremarkable. However, elevated levels of lactic dehydrogenase (520 UI/L) and Beta 2-microglobulin (4.2; reference values 0-3) were noted. Viral hepatitis B, C, and HIV tests returned negative. The patient's karyotype was normal, and her myelogram was normocellular, showing no increased mature lymphocytes or morphological alterations. An upper gastrointestinal endoscopy ruled out *H. pylori* infection or malignancy.

**Fig. 1:**
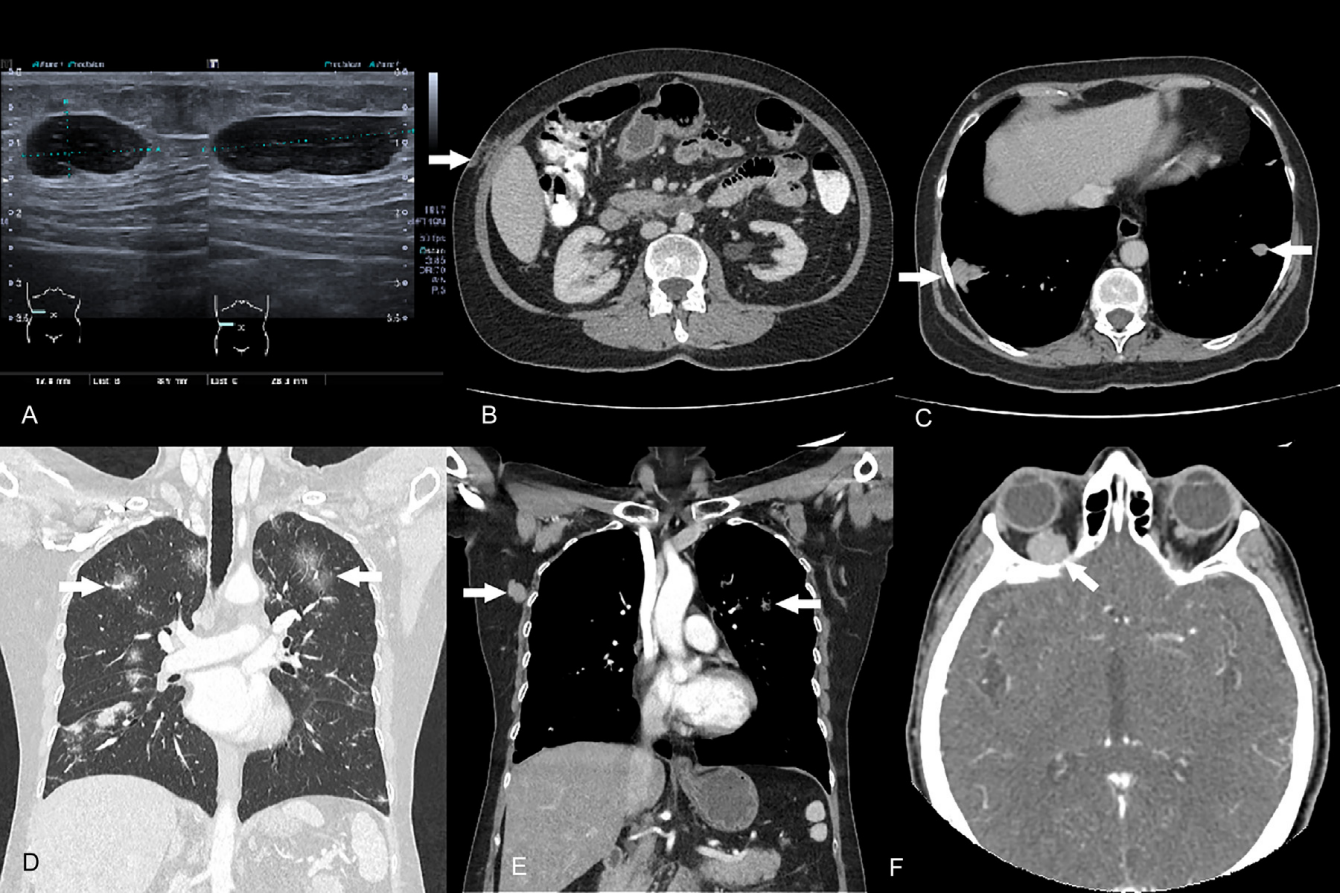
Demonstrative findings highlighted with white arrows. (A) Ultrasound of the soft tissues in the right hypochondrium displaying hypoechoic subcutaneous nodules with distinct borders and minimal flow on color Doppler. (B) Contrast-enhanced tomography of the chest with a coronal window reconstruction for the lungs, showcasing multiple central and peripheral subsolid nodules with a ground glass halo effect. (C) Mediastinal window reveals right axillary adenomegaly with no other lymph node involvement detected. (D) Contrast tomography of the abdomen shows subcutaneous nodules in the right hypochondrium region. (E) The lower base of the thorax displays solid pulmonary and subcutaneous nodules. (F) Neck tomography highlights an orbital soft tissue mass with right intraconal enhancement encompassing the optic nerve.

**Fig. 2:**
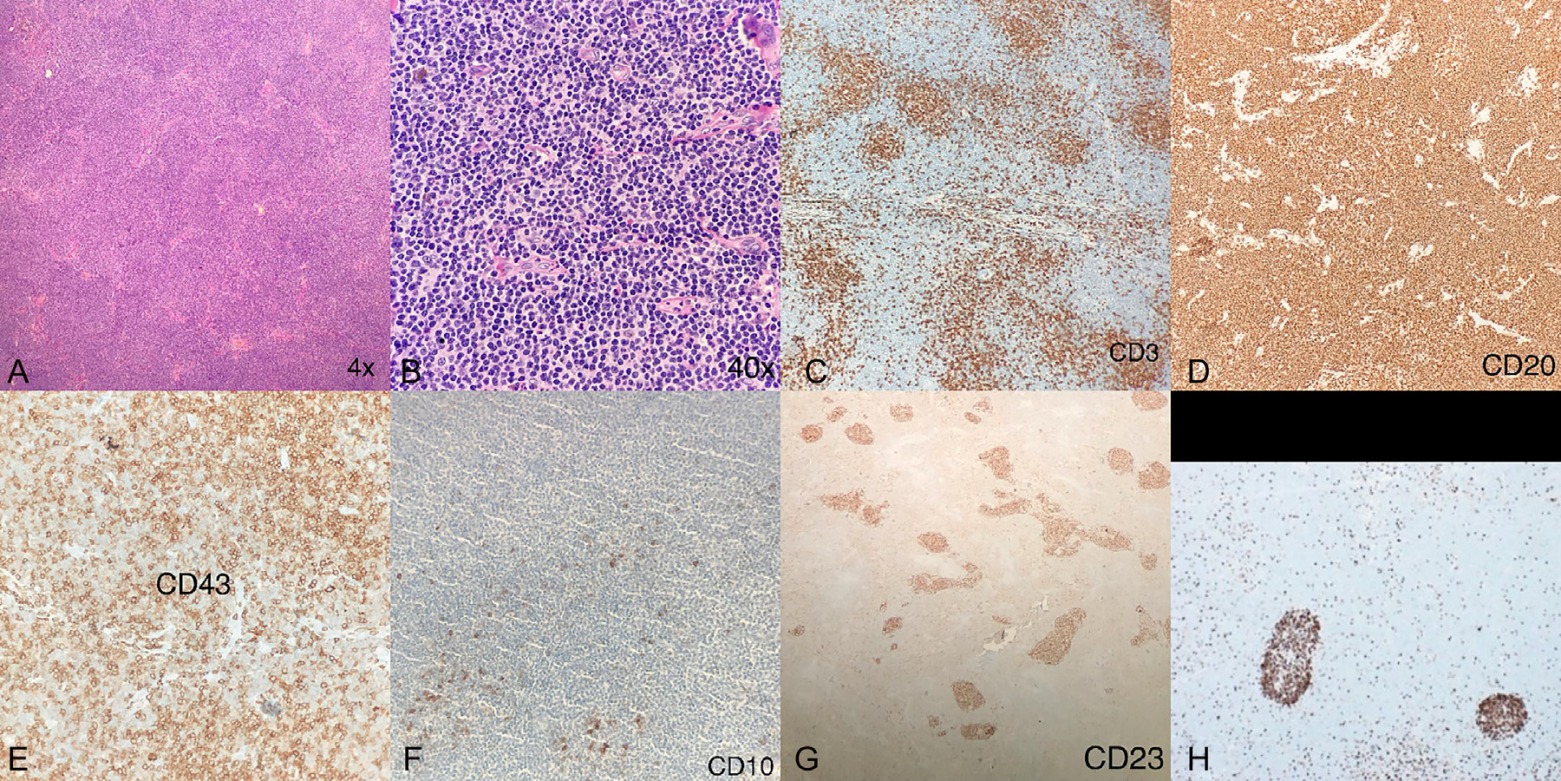
Trucut biopsy of the subcutaneous cell tissue lesions shows: (A) Hematoxylin-eosin (4×): Diffuse cell infiltrate. (B) Hematoxylin-eosin (40×): Lymphocytes present. (C) CD3: Negative. (D) CD20: Positive. (E) BCL2: Positive. (F) CD43: Positive. (G) BCL6: Negative. (H) Ki-67: 40%.

Abdominal ultrasound revealed normal internal organs. Nevertheless, in the soft tissues of the right hypochondrium, a hypoechoic, hypovascular, solid subcutaneous nodule with well-defined borders was identified. A TRUCUT biopsy of this nodule was conducted (Fig. 1). Histopathologic examination revealed type B lymphoid cells that were positive for CD20, CD43, and BCL 2 and negative for CD5, CD10, and CD23. Proliferative activity was up to 40% (Ki 67), leading to a diagnosis of Marginal zone B cell lymphoma (Fig. 2).

Contrast-enhanced CT scans of the neck, thorax, and abdomen were then performed. Findings included bilateral intraconal involvement with masses and solid nodules of the orbital soft tissues and optic nerves. The ocular adnexa appeared normal. In the thorax, both central and peripheral solid and subsolid pulmonary nodules were seen, surrounded by a ground-glass halo; there was no enlargement of mediastinal or axillary lymph nodes. The abdomen exhibited solid subcutaneous nodules in the right hypochondrium, lumbar region, and left gluteus, as well as nodules in the mesentery and retroperitoneum. No lymphadenopathy, hepatomegaly, or splenomegaly were observed (Fig. 1).

**Fig. 3:**
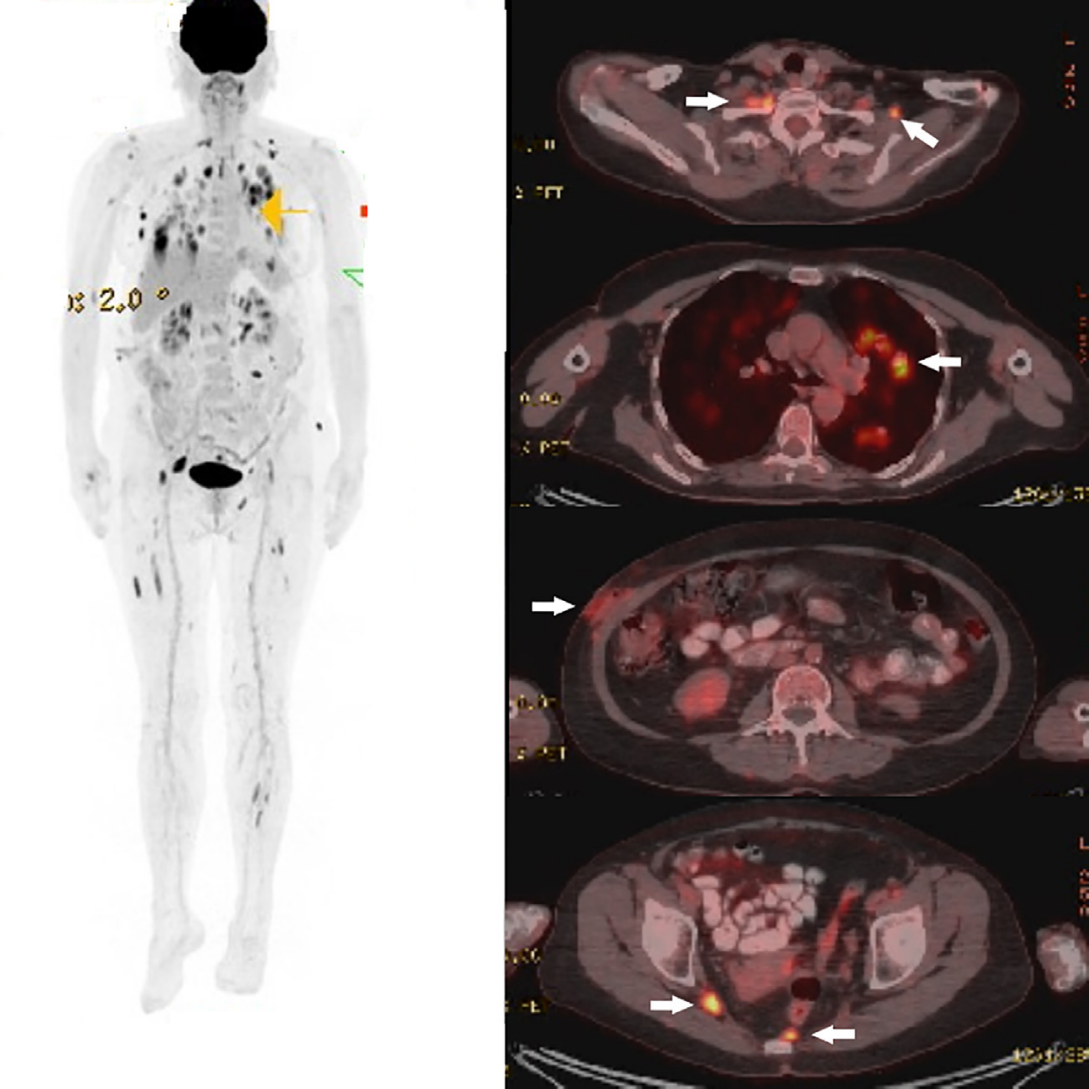
Key findings are emphasized with white arrows. Positron emission tomography depicts areas of heightened metabolism in the posterior cervical and supraclavicular lymph nodes. Multiple solid and subsolid pulmonary nodules are hypermetabolic without evident size enlargement. Subcutaneous nodules are observed in the right hypochondrium, lumbar region, left gluteal, as well as perirenal, left mesenteric, presacral, left obturator, and perirectal areas.

A subsequent 18-fluoro-deoxyglucose (FDG) positron emission tomography (PET) scan showed hypermetabolic cervical adenopathy, particularly in the posterior cervical and left supraclavicular regions, without any lymph node enlargement. The previously described nodules in the abdomen and thorax were also hypermetabolic, alongside other nodes showing increased uptake at the perirenal, left obturator, presacral, and perirectal sites (Fig. 3).

These findings solidified the diagnosis of stage IV extranodal B lymphoma of the marginal zone, characterized by multiorgan involvement [2]. Under the MALT International Prognostic Index (MALT IPI) classification, when considering prognostic risk factors for EMZL (such as age >70 years, stage III/IV, and elevated LDH), this patient was categorized in the high-risk group, accruing three points [2].

In response to her diagnosis, the patient underwent treatment with Rituximab and Bendamustine. She responded favorably, and subsequent follow-ups revealed a marked reduction and eventual disappearance of the identified lesions.

## Discussion

Extranodal manifestations of lymphoma can occur in any organ, being more common in non-Hodgkin's than in Hodgkin's disease. Typical lymphoma findings encompass generalized lymphadenopathy or nodal conglomerates/masses; soft tissue masses showing limited local destruction; single or multiple masses in solid organs; and diffuse thickening of the intestinal wall without obstruction [6].

EMZL originates in acquired lymphoid tissue typically subjected to prolonged antigenic stimulation. While the cause of MALT development can vary depending on the location, it is generally attributed to chronic infections or autoimmune disorders when determined [7].

The clinical manifestations of EMZL are organ-dependent. Consequently, symptoms vary among subtypes: cutaneous EMZL presents as skin nodules or papules, pulmonary EMZL often manifests as recurrent respiratory infections, gastric EMZL may produce occult bleeding and dyspepsia, and ocular EMZL typically results in symptoms like red eyes, excessive tearing, or visual field abnormalities [5]. Pulmonary EMZL might show up as recurrent respiratory infections or be revealed through imaging as pulmonary nodules, while the ocular variant could display as redness or a slowly growing mass in ocular adnexa [7,8].

Hence, EMZL should be consistently featured in differential diagnoses when encountering lesions like nodules, masses, or mucosal thickening. Radiologists should familiarize themselves with the diagnostic imaging findings of EMZL to discern it from other potential diagnoses [9].

Confirmatory diagnosis leans heavily on histopathologic identification. Features include marginal zone B cells with diffuse infiltration alongside seemingly reactive follicles, and immunophenotypically, cells testing positive for B markers like CD19, CD20, and CD22, and negative for CD5, CD10, and CD23 [3,5,8,10].

The differential diagnoses of NHL through imaging span a wide array of lesions—benign to malignant, infectious to autoimmune, and primary tumors; contingent on their anatomical sites. However, recognizing the typical imaging presentations of extranodal lymphoma facilitates precise diagnosis [9,10].

The MALT International Prognostic Index (MALT IPI) serves as a valuable prognostic tool, incorporating parameters such as age (>70 years), advanced staging (III or IV), and raised LDH levels. Based on these criteria, patients are stratified into low, intermediate, and high-risk categories. Correspondingly, the 5-year event-free survival (EFS) rates for these classifications stand at 70%, 56%, and 29% respectively [5].

Treatment strategies for EMZL are contingent on gastric involvement. In cases lacking gastric compromise, localized disease might be addressed with first-line ISRT, though the optimal dosage remains a topic of debate. Cutaneous EMZL, being largely confined to the skin with only a minor proportion (6%) exhibiting extracutaneous dissemination, often sees symptomatic patients or those in advanced stages being treated with rituximab as a monotherapy, given the indolent progression of the condition [[5], [11], [12]].

If gastric involvement is detected alongside *H. pylori* presence, the initial step involves antibiotic administration to counter antigenic stimulation, followed by a 3-month endoscopic re-evaluation. Current guidelines advocate a watchful waiting approach for a minimum of 12 months post-treatment before considering further therapeutic interventions. In symptomatic patients, or those in advanced stages who have not responded favorably to anti-*H. pylori* or ISRT therapy, immunotherapy and chemoimmunotherapy stand as the preferred choices [[2], [8], [11], [12]]. Consensus regarding treatment approaches for patients experiencing relapse or demonstrating resistance remains elusive [5].

## Conclusion

EMZL presents as a heterogeneous disease, notable for its variable clinical manifestations, indolent progression, and extended survival rates [12]. Diagnostic imaging remains pivotal in delineating the disease based on the affected organ(s), demarcating its extent, guiding biopsy procedures, and facilitating follow-up. Consequently, EMZL ought to be considered in differential diagnoses for lesions manifesting as nodules, masses, or mucosal thickening. It is imperative for radiologists to be well-acquainted with these specific findings to inform their differential diagnoses accurately [4,5].

## Patient consent

I, Walter Camilo Mera Romo, author of the case report “Extranodal marginal zone lymphoma: A case report” reported that the informed consent for publication was authorized in writing by the patient.
